# The Premonitory Urge for Tics Scale in a large sample of children and adolescents: psychometric properties in a developmental context. An EMTICS study

**DOI:** 10.1007/s00787-019-01450-1

**Published:** 2019-12-04

**Authors:** Thaïra J. C. Openneer, Zsanett Tárnok, Emese Bognar, Noa Benaroya-Milshtein, Blanca Garcia-Delgar, Astrid Morer, Tamar Steinberg, Pieter J. Hoekstra, Andrea Dietrich, and the EMTICS collaborative group, Alan Apter, Valentina Baglioni, Juliane Ball, Noa Benaroya-Milshtein, Benjamin Bodmer, Emese Bognar, Bianka Burger, Judith Buse, Francesco Cardona, Marta Correa Vela, Nanette M. Debes, Andrea Dietrich, Maria Cristina Ferro, Carolin Fremer, Blanca Garcia-Delgar, Mariangela Gulisano, Annelieke Hagen, Julie Hagstrøm, Tammy J. Hedderly, Isobel Heyman, Pieter J. Hoekstra, Chaim Huyser, Marcos Madruga-Garrido, Anna Marotta, Pablo Mir, Astrid Morer, Norbert Müller, Kirsten Müller-Vahl, Alexander Münchau, Peter Nagy, Valeria Neri, Thaïra J.C. Openneer, Alessandra Pellico, Kerstin J. Plessen, Cesare Porcelli, Marina Redondo, Renata Rizzo, Veit Roessner, Daphna Ruhrman, Jaana M.L. Schnell, Paola Rosaria Silvestri, Liselotte Skov, Tamar Steinberg, Friederike Tagwerker Gloor, Zsanett Tárnok, Jennifer Tübing, Victoria L. Turner, Frank Visscher

**Affiliations:** 1 Department of Child and Adolescent Psychiatry, University of Groningen, University Medical Center Groningen, Hanzeplein 1 XA10, 9713 GZ Groningen, The Netherlands; 2 Vadaskert Child and Adolescent Psychiatric Hospital, Budapest, Hungary; 3 Child and Adolescent Psychiatry Department, Affiliated to Sackler Faculty of Medicine, Schneider Children’s Medical Center of Israel, Tel Aviv University, Petah-Tikva, Israel; 4 Department of Child and Adolescent Psychiatry and Psychology, Institute of Neurosciences, Hospital Clinic Universitari, Barcelona, Spain; 5 Institut d’Investigacions Biomediques August Pi i Sunyer (IDIBAPS), Barcelona, Spain; 6 Centro de Investigacion en Red de Salud Mental (CIBERSAM), Instituto Carlos III, Madrid, Spain

**Keywords:** Tourette syndrome, Premonitory urges, Premonitory Urge for Tics Scale (PUTS), Psychometric properties, Obsessive–compulsive symptoms

## Abstract

Premonitory urges are uncomfortable physical sensations preceding tics that occur in most individuals with a chronic tic disorder. The Premonitory Urge for Tics Scale (PUTS) is the most frequently used self-report measure to assess the severity of premonitory urges. We aimed to evaluate the psychometric properties of the PUTS in the largest sample size to date (*n *= 656), in children aged 3–16 years, from the baseline measurement of the longitudinal European Multicenter Tics in Children Study (EMTICS). Our psychometric evaluation was done in three age-groups: children aged 3–7 years (*n *= 103), children between 8 and 10 years (*n *= 253), and children aged 11–16 years (*n *= 300). The PUTS exhibited good internal reliability in children and adolescents, also under the age of 10, which is younger than previously thought. We observed significant but small correlations between the severity of urges and severity of tics and obsessive–compulsive symptoms, and between severity of urges and ratings of attention-deficit/hyperactivity disorder and internalizing and externalizing behaviors, however, only in children of 8–10 years. Consistent with previous results, the 10th item of the PUTS correlated less with the rest of the scale compared to the other items and, therefore, should not be used as part of the questionnaire. We found a two-factor structure of the PUTS in children of 11 years and older, distinguishing between sensory phenomena related to tics, and mental phenomena as often found in obsessive–compulsive disorder. The age-related differences observed in this study may indicate the need for the development of an age-specific questionnaire to assess premonitory urges.

**Electronic supplementary material:**

The online version of this article (10.1007/s00787-019-01450-1) contains supplementary material, which is available to authorized users.

## Introduction

Chronic tic disorders, i.e. Tourette syndrome (TS) and persistent (chronic) motor or vocal tic disorder, are childhood-onset disorders characterized by the presence of multiple motor and/or vocal tics for at least 1 year [1]. Tic disorders are often accompanied by other disorders, particularly obsessive–compulsive disorder (OCD) and attention-deficit/hyperactivity disorder (ADHD), but also autism spectrum disorder (ASD) and internalizing problems (i.e. anxiety or depression) [2].

Up to 93% of individuals with TS experience an uncomfortable physical sensation preceding their tics, known as a premonitory urge [3]. Two broad types of premonitory urges have been reported: sensory feelings such as an ‘itch’ or ‘pressure’ in certain bodily areas, or mental phenomena such as ‘the feeling that something is not “just right” or complete’ [4, 5]. Premonitory urges are often reported to be even more distressing and impairing than tics themselves [6, 7] and are an important target for behavioral therapy [8, 9], as they may facilitate suppression of the impending tic. In recent years, our understanding of the premonitory urge in TS has rapidly expanded (see for a review [4]), providing more knowledge about the role of premonitory urges in TS. For example, the level of interoceptive awareness proved to be one of the stronger predictors of premonitory urges in TS [43].

Despite the recent advances in our understanding of the role of premonitory urges in TS, there is still much uncertainty about the age of onset and development of premonitory urges across childhood and adolescence. For instance, while tics typically start around the age of 6–7 years, it has been assumed that children do not become aware of their premonitory urges until on average 3 years after tic onset [3, 10]. This suggests that premonitory urges may not be present at the onset of TS, but may develop later [11, 12]. In addition, it has been thought that young children are less consistent in reporting their awareness of premonitory urges before the age of 10 years [12]. However, a recent large study found that premonitory urges were reported in 46.7% of the children with TS younger than 10 years, thus suggesting that premonitory urges may be experienced at a younger age than previously thought and, furthermore, that children under the age of 10 may be able to reliably report their premonitory urges [14].

The Premonitory Urge for Tics Scale (PUTS [12]) is the most frequently used self-report measure to assess the severity of premonitory urges. Studies investigating the psychometric properties of the PUTS have so far indicated a good internal reliability and correlations with the Yale Global Tic Severity Scale (YGTSS [15]) for children of 11 years and older, but not for younger children [12, 16, 17]. Similarly, PUTS scores of children aged 11 years and older (and not younger children) correlated well with the Children’s Yale-Brown Obsessive Compulsive Scale (CY-BOCS [18]), which might not be surprising given that some premonitory urges (i.e. ‘the feeling that something is not “just right” or not complete’) have been shown to be related to OCD symptoms [5]. Thus, while studies so far observed good psychometric properties of the PUTS in children of 11 years and older [12, 16, 17], the suitability of the PUTS for younger children has not yet been established, even though premonitory urges may already be present at a young age.

The PUTS was originally designed as a one-dimensional measure [12]. However, a two- to three-factor [16, 19] solution emerged from recent factor analyses in adolescents and adults; one factor broadly represented mental urges, including the aforementioned OCD-related premonitory urges, i.e. ‘the feeling that something is not “just right” or not complete’ [19], while the second factor reflected the intensity or frequency of the urges [16]. Yet, given that the typical course of TS is characterized by a symptomatic peak in early adolescence and decline into adulthood [20], findings from adolescents and adults may not hold true for younger children. Furthermore, existing studies examining the psychometric properties of the PUTS in children and adolescents are hampered by small sample sizes (*n *= 40 to *n *= 82; [12, 16, 17]), which made it difficult to investigate age-related differences in the psychometric properties of the PUTS across childhood and adolescence.

The aim of the present study, therefore, was to examine the psychometric properties of the PUTS in a large sample of 656 children, aged 3–16 years (of which 356 children were below 11 years) from an European multicenter study. We aimed to replicate previous work [12, 16, 17] and to further investigate the psychometric properties in young children. First, we investigated the internal consistency of the PUTS. Second, we assessed correlations with tic and OCD severity, also exploring the influence of two OCD-related items of the PUTS. Third, we looked into associations of the PUTS with other comorbid symptom domains (i.e. ADHD, oppositional defiant disorder [ODD], ASD, and externalizing and internalizing symptoms), given the previous inconsistent literature in small samples [5, 12, 16, 21]. Finally, to extend earlier work [16, 19] we conducted a factor analysis of the PUTS in the whole sample and in three different age groups.

## Methods

### Participants

Our study sample consisted of 656 3–16 years old children and adolescents with a chronic tic disorder participating in the baseline measurement of the longitudinal European Multicenter Tics in Children Study (EMTICS). EMTICS aims to identify the role of genes, autoimmunity, and psychosocial stress on the onset and course of tics (see for a more detailed description: [22]). Participants were recruited from 16 child and adolescent psychiatry or pediatric neurology outpatient clinics, or through advertisement of the study to patient organizations and other health professionals. Exclusion criteria were having a serious medical illness, treatment with antibiotics during the last month (as the included children were also eligible to participate in a separate antibiotic study [see 22]), or an inability to understand and comply with the study procedures. The adolescent’s parents or legal guardians provided written informed consent and the participating adolescent provided written consent or assent in line with the local medical-ethical regulations. The study was approved by the local research ethics committee of the participating centers.

### Procedures

Children and adolescents were asked to complete questionnaires on premonitory urges and symptoms of ADHD, ODD, ASD, and internalizing and externalizing disorders within 2 weeks before the baseline visit, and to bring these to the first visit. During the baseline visit a trained study clinician assigned a clinical diagnosis of a chronic tic disorder, OCD, and/or ADHD according to DSM-IV-TR criteria [13], and rated the severity of tics and OCD with well-validated measures (see further below).

### Measures

#### Premonitory Urge for Tics Scale (PUTS)

The PUTS was developed by Woods et al. [12] and has previously been demonstrated as having good internal reliability, temporal stability, and correlations with the YGTSS and CY-BOCS in children of 11 years and older and in adults [12, 16, 17, 19, 23]. It measures sensory and mental phenomena associated with premonitory urges in 10 items on a four-point scale (range 10–40). The first 6 items include itchiness, energy, pressure, tense feeling, incomplete, or a not “just right” feeling before performing a tic. The additional 4 items assess whether these feelings are experienced almost all the time before a tic, if they happen with every tic, if they go away after the tic is performed, and if subjects are able to stop the tics for a short period of time. Woods et al. [12] noted that the 10th item had a lower correlation with the rest of the scale compared to the other items. As a result, some studies using the PUTS omit the 10th item in favor of a 9-item scale (e.g., [17]. In the present study, the 10-item PUTS was administered to participants to replicate the data analysis of Woods et al. [12] (i.e., to determine how the 10th item correlated with the rest of the scale using a larger sample size). A higher total score indicates more severe premonitory urges.

#### Yale Global Tic Severity Scale (YGTSS)

The YGTSS [15] (Cronbach’s alpha in our study *α* = 0.87) is a semi-structured clinician-rated instrument that evaluates the severity of tics across five dimensions each scored on a five-point scale, by assessing the number, frequency, intensity, complexity, and interference of, respectively, motor and vocal tics during the past week. A total tic severity score can be obtained (range 0–50), and also severity scores for vocal tics (range 0–25, *α* = 0.85) and motor tics (range 0–25, *α* = 0.89) by summing up the respective scores. A higher total, vocal, or motor score indicates more severe tics.

#### Children’s Yale-Brown Obsessive–Compulsive Scale (CY-BOCS)

The CY-BOCS is a clinician-administered semi-structured interview developed to assess the severity of obsessions and compulsions in children [18, 24] (Cronbach’s alpha in our study *α* = 0.93). The symptoms are evaluated across five areas, including the time, interference, distressing nature, resistance, and control associated with obsessions and compulsions during the past week on a five-point scale. Besides a total OCD severity score (range 0–40), a severity score was obtained for obsessions (range 0–20; *α* = 0.92) and compulsions (range 0–20; *α* = 0.94). A higher score indicates higher severity ratings.

#### Other symptom domains

To assess ADHD and ODD symptom severity, we used the parent-rated Swanson Nolan and Pelham-IV rating scale (SNAP-IV [25, 26]). To investigate ASD severity, we used the parent-rated Autism Spectrum Screening Questionnaire (ASSQ [27]), while the Strengths and Difficulties Questionnaire (SDQ [28]) was used to assess broadband internalizing and externalizing symptom severity. See Supplement 1 for more information about these questionnaires.

### Data analytic strategy

Prior to analysis, we removed outliers (≥ |3.0| standard deviations from the mean; up to 0.9%). We checked on the normal distribution of the residues, and used log-transformation to normalize scale scores where appropriate (i.e., only for the total severity score of the CY-BOCS, leading to a normal distribution). Then, site differences were removed by regressing out the effect of site variance from each measure and the saved residuals were added to each score of the respective variable that was used for analysis.

We distinguished three age groups: children ≤ 7 years (*n *= 103), children between 8 and 10 years (*n *= 253), and children ≥ 11 years (*n *= 300). As a supplementary analysis to allow for comparisons with the existing literature [12, 16, 17], we also divided our sample into two age groups; children ≤ 10 years, (*n *= 356) and children and adolescents ≥ 11 years, (*n *= 300).

Between-group characteristics were tested with the non-parametric Kruskal–Wallis H test (as sex was non-normally distributed), a Chi-square (*χ*^2^) test, and an analysis of variance (ANOVA), with a Bonferroni correction for multiple comparisons. Differences in the means of the PUTS total score and individual PUTS items between different age groups were also tested with a Bonferroni-corrected ANOVA. For each age group, the Cronbach’s alpha (*α*) was first calculated for the 10 PUTS items, and additionally for the 9-item PUTS omitting the 10th item to determine internal reliability. In addition, the item-total correlation (i.e. the correlation between each individual item and the remaining items) was evaluated by Pearson’s product-moment correlation coefficients (*r*); *r* values > 0.20 were considered satisfactory [29]. In addition, the Cronbach’s α was calculated over the remaining items (thus, without the initial individual items). A Cronbach’s *α* value of around 0.7 was considered acceptable, of 0.8 good, and of 0.9 excellent [30].

To examine the correlations between the PUTS and tic and OCD severity, Pearson product–moment correlations were computed. We additionally explored correlations of the PUTS with symptom severity of ADHD, ODD, ASD, and internalizing and externalizing symptoms. Effect sizes between 0.1 and 0.3 were considered low, between 0.3 and 0.5 moderate, and those over 0.5 high [31].

Furthermore, the underlying factor structure of the PUTS was investigated by conducting a principal axis exploratory factor analysis (EFA). We used direct oblimin rotation, as we assumed that possible factors would be correlated in line with a previous study [16], first, for the total group, and then for different age groups. The factorability of the data (i.e. the assumption that there are correlations amongst items so that coherent factors can be identified), was tested by looking at the inter-item correlations and measures of sampling adequacy. Ideally, an inter-item correlation matrix is considered factorable when the majority of the correlation coefficients computed are in the moderate range; i.e. *r* values between .20 and .80 [32]. If an item produced a significant number (two or more) inter-item correlations below .20 (i.e., items are not representing the same construct) or above .80 (i.e., multicollinearity), the items were removed before conducting the EFA [32, 33]. The adequacy of the sampling for the factor analysis with the remaining items was estimated with the Kaiser–Meyer–Olkin (KMO) statistic; its values range from 0 to 1. KMO values greater than 0.6 represent acceptable sampling adequacy [34]. In addition, Bartlett’s test of sphericity was used to assess the suitability of the data for structure detection: a significant test indicates that the individual variables are sufficiently correlated for a factor analysis to be performed. As an outcome measure, we looked at the communalities, representing the proportion of the variance that can be accounted for by the extracted factors. Number of factors were determined by the scree plot and eigenvalues > 1 [33]. Low communality scores < 0.02 may indicate that there are additional factors, which thus should be removed from the current factor [32].

Finally, as a sensitivity analysis, we re-analyzed the correlations between the PUTS and CY-BOCS and the factor analyses without the two OCD-related items (i.e. items 4 and 5: ‘the feeling that something is not “just right” or not complete’), and repeated all analyses without excluding outliers. All statistical analyses were performed using SPSS version 23 (SPSS Inc. USA), using a significance level of *p* < 0.05.

## Results

### Group characteristics

See Table 1 for the group characteristics. The mean age for tic onset in the total sample was 6 years. Children aged ≤ 7 years experienced the least amount of urges (81%), whereas children aged ≥ 11 years reported the most urges (97.5%). All age groups differed significantly from each other in PUTS severity; children ≤ 7 years had the lowest PUTS severity score, and children ≥ 11 years the highest score. Children ≥ 11 years had higher tic severity as measured by the YGTSS compared to children of ≤ 7 years, but not to children 8–10 years. There were no significant age group differences in sex, OCD severity, or presence of comorbid OCD or ADHD diagnoses, although comorbid OCD and ADHD diagnoses increased (non-significantly) across age.

**Table 1:** Group characteristics

	Total sample (*n *= 656)	Children ≤ 7 (*n* = 103)	Children 8–10 (*n* = 253)	Childre*n* ≤ 10 (*n* = 356)	Childre*n* ≥ 11 (*n* = 300)	Test statistic	
Male sex, *n* (%)	498 (75.9)	77 (74.8)	189 (74.7)	266 (74.7)	232 (77.3)	*χ*^2^= 0.61^a^	
Children with premonitory urges, %	93.7	81	95.5	90.8	97.5	*χ*^2^=133.49**^a^	Children ≤ 7 **< **Children 8–10 Children ≤ 7 **< **Children ≥ 11 Children 8–10 **< **Children ≥ 11 Children ≤ 10 **< **Children ≥ 11
Premonitory urges severity, *M *± SD (range)	20.16 ± 6.17 (10–38)	17.03 ± 6.15 (10–30)	19.40 ± 6.14 (10–37)	18.72 ± 6.20 (10–37)	21.87 ± 5.65 (10–38)	*F*(2653) = 29.02**^b^	Children ≤ 7 **< **Children 8–10 Children ≤ 7 **< **Children ≥ 11 Children 8–10 **< **Children ≥ 11 Children ≤ 10 **< **Children ≥ 11
Tic onset, years, *M *± SD	6.03 ± 2.19	4.61 ± 1.05	5.70 ± 1.79	5.38 ± 1.68	6.81 ± 2.46	*F*(2520) = 39.45**^b^	Children ≤ 7 **< **Children 8–10 Children ≤ 7 **< **Children ≥ 11 Children 8–10 **< **Children ≥ 11 Children ≤ 10 **< **Children ≥ 11
Tic severity, *M *± SD (range)	19.68 ± 8.67 (0–44)	17.67 ± 8.74 (0–35)	19.39 ± 8.45 (0–41)	18.96 ± 8.57 (0–41)	20.54 ± 8.71 (0–44)	*F*(2653) = 4.37*^b^	Children ≤ 7 **< **Children ≥ 11
OCD severity, *M *± SD (range)	6.54 ± 8.57 (0–36)	5.35 ± 7.48 (0–30)	6.02 ± 8.54 (0–34)	5.83 ± 8.24 (0–34)	7.38 ± 8.88 (0–36)	*F*(2650) = 2.88^b^	
Comorbid OCD diagnosis, *n* (%)	178 (27.1)	20 (19.4)	66 (26.1)	85 (23.9)	93 (31)	*F*(2650) = 1.92^b^	
Comorbid ADHD diagnosis, *n* (%)	186 (28.4)	22 (21.4)	67 (26.5)	89 (25)	97 (32.3)	*F*(2652) = 2.61^b^	

Premonitory urges assessed by the 10-item Premonitory Urge for Tics Scale [12]; Tic severity assessed by the Yale Global Tic Severity Scale (YGTSS [15]); CY-BOCS severity assessed by the Children’s Yale-Brown Obsessive Compulsive Scale (CY-BOCS [18]); Note that in the total sample 71 participants (10.8%) had both a comorbid ADHD and OCD diagnosis according to DSM-IV-TR criteria

Between-group differences were tested by ^a^Pearson’s Chi-squared test and ^b^Analysis of Variance; *p < 0.05 **p < 0.001

### Item-by-item frequencies of the PUTS

Table 2 shows that the group of children ≥ 11 years had the highest mean scores on most items of the PUTS, the children between 8 and 10 years scored intermediate, and the youngest group (≤ 7 years) scored lowest. Likewise, in the two-group analysis, children ≥ 11 years had higher mean PUTS scores per individual PUTS item compared to children ≤ 10 years, except for item 1 and 4 (see Supplementary Table S2a).

**Table 2:** Comparison of individual PUTS items between children of different age groups: means, standard deviations, item-total correlations (Pearson’s *r*) and internal reliability (Cronbach’s *α*)

	Group 1: Children ≤ 7 years (*n* = 103)				Group 2: Children 8–10 years (n = 253)				Group 3: Children ≥ 11 years (n = 300)				Test statistic	
	Mean	SD	Pearson’s *r*	*α* if item removed	Mean	SD	Pearson’s *r*	*α* if item removed	Mean	SD	Pearson’s *r*	*α* if item removed		
PUTS 1	1.40	0.73	0.40**	0.84	1.63	0.95	0.42**	0.80	1.64	0.94	0.20**	0.74	*F*(2654) = 2.86	
PUTS 2	1.53	0.95	0.52**	0.83	1.66	0.93	0.56**	0.78	1.89	1.01	0.42**	0.71	*F*(2653) = 6.11*	Group 1 < Group 3 Group 2 < Group 3
PUTS 3	1.59	0.91	0.50**	0.82	1.84	1.00	0.46**	0.79	2.06	1.00	0.42**	0.71	*F*(2637) = 9.16**	Group 1 < Group 3 Group 2 < Group 3
PUTS 4	1.63	0.94	0.46**	0.83	1.64	0.92	0.50**	0.79	1.79	1.00	0.41**	0.71	*F*(2650) = 2.18	
PUTS 5	1.43	0.91	0.48**	0.82	1.56	0.88	0.43**	0.80	1.72	1.04	0.43**	0.71	*F*(2652) = 4.17*	Group 1 < Group 3
PUTS 6	1.76	1.08	0.62**	0.82	2.09	1.11	0.50**	0.79	2.38	1.10	0.42**	0.71	*F*(2655) = 13.01**	Group 1 < Group 2 Group 1 < Group 3 Group 2 < Group 3
PUTS 7	1.90	1.03	0.73**	0.81	2.18	1.12	0.72**	0.76	2.60	1.11	0.62**	0.68	*F*(2653) = 18.89**	Group 1 < Group 3 Group 2 < Group 3
PUTS 8	1.86	1.11	0.62**	0.82	1.99	1.05	0.54**	0.78	2.30	1.12	0.53**	0.69	*F*(2653) = 8.95**	Group 1 < Group 3 Group 2 < Group 3
PUTS 9	2.12	1.16	0.61**	0.82	2.39	1.22	0.60**	0.78	2.66	1.15	0.44**	0.71	*F*(2653) = 9.39**	Group 1 < Group 3 Group 2 < Group 3
PUTS 10	2.07	1.09	0.40**	0.84	2.58	1.07	0.16*	0.83	3.00	1.00	0.06	0.76	*F*(2646) = 33.05**	Group 1 < Group 2 Group 1 < Group 3 Group 2 < Group 3
*α* 9-items	0.84				0.83				0.76					
*α* 10-items	0.81				0.79				0.72					

*PUTS* Premonitory Urge for Tics Scale [12], each item scored on a 4-point scale from 1 = ‘not at all true’ to 4 = ‘very much true’. See Table S2a for results comparing children ≤ 10 years and ≥ 11 years, where mean PUTS scores differed significantly for all items, except item 1 and 4. *α* 9-items indicated the Cronbach’s *α* for item 1–9 of the PUTS, whereas *α* 10-items indicates the Cronbach’s *α* for item 1–10 of the PUTS

Between-group differences were tested by an Analysis of Variance; *p *< 0.05; **p < 0.001

**Fig. 1:**
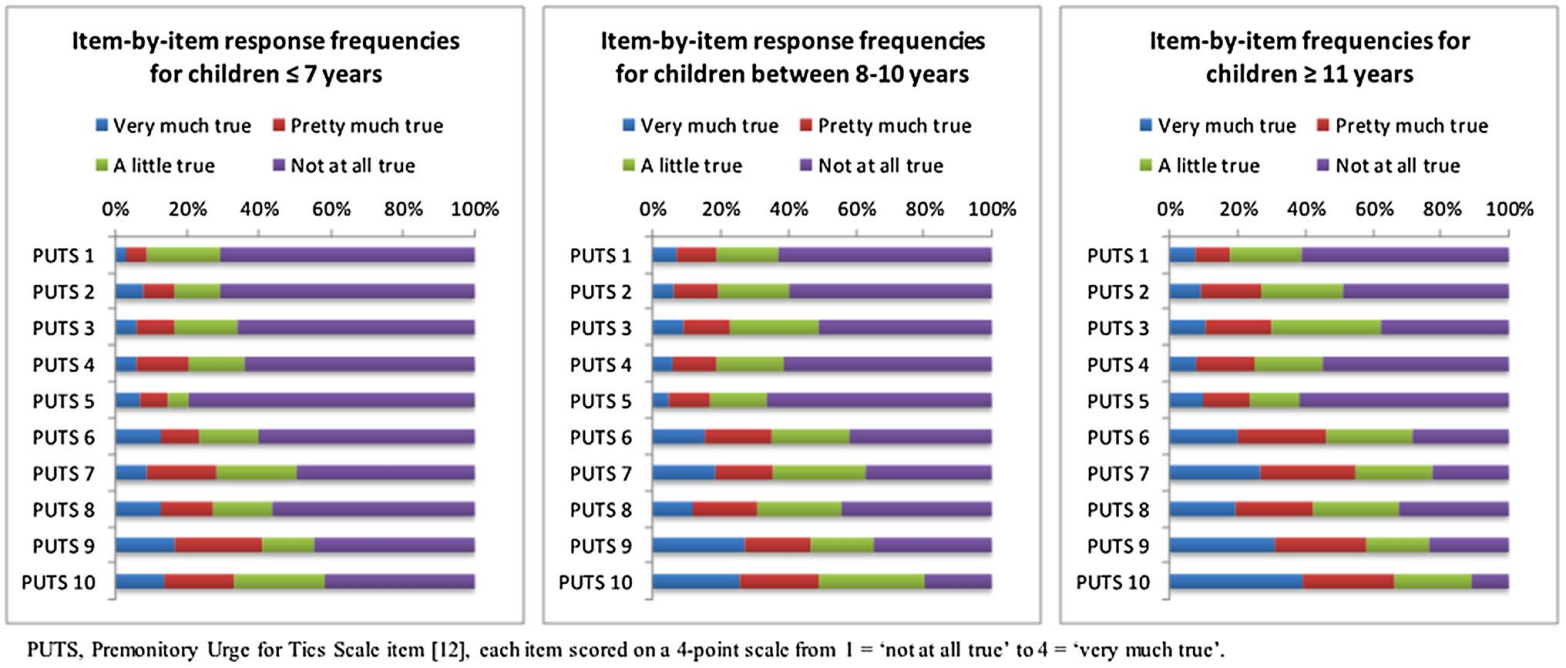
Item-by-item response frequencies of premonitory urges for children of 7 years and younger, children between 8 and 10 years and children of 11 years and older

See Fig. 1 for item-by-item response frequencies of the PUTS for children in the three age groups. Items 1–3 were on average reported by 20% of the children ≤ 7 years, 30% of children 8–10 years, and 40% of children ≥ 11 years. The most commonly endorsed sensations in all groups were items 6–10, from 40% of the children ≤ 7 years to 70% of the children ≥ 11 years. The OCD-related urges ‘feelings of something being not “just right” or not complete’ (items 4 and 5) were endorsed by almost 40% and 20% of children ≤ 7 years; 40% and 30% of children between 8 and 10; and 45% and 40% children ≥ 11 years, respectively.

### Means, standard deviations, internal reliability

Table 2 presents the Cronbach’s *α* for each PUTS item across the three age groups after removal of the respective item. Consistent with the decision of previous authors [12, 16] to remove item 10 from further analyses, the results showed a lower correlation of item 10 with the rest of the scale for all age groups relative to the other items. Furthermore, the Cronbach’s *α* was similar or higher for all age groups after omitting the 10th item. Therefore, the subsequent analyses were done with the first 9 items of the PUTS. Thus, for the total sample of 656 children, the Cronbach’s *α* for the 9-item PUTS was .80 (*α* = 0.78 for the 10-item PUTS), representing good internal reliability. (See Supplementary Table S2a for the Cronbach’s α for each PUTS item in the two-group analysis).

### Associations of the PUTS with the YGTSS and CY-BOCS

For the total sample of 656 children, we observed significant but small positive correlations between the PUTS and the YGTSS total score and all subscales (see Table 3). After analyzing the three age groups, we found that children aged 8–10 years old drove the significant correlations, but not younger or older children. Similarly, in the two-group analysis, significant correlations were present only in children ≤ 10 years and not in children ≥ 11 years (Supplementary Table S2b).

**Table 3:** Correlations between the PUTS total score and the YGTSS and CY-BOCS scales for the total sample and different age groups

	YGTSS total score	YGTSS motor tics	YGTSS motor tic dimensions					YGTSS vocal tics	YGTSS vocal tic dimensions				
		Subscale score	Number	Frequency	Intensity	Complexity	Interference	Subscale score	Number	Frequency	Intensity	Complexity	Interference
Total sample (*n *= 656)	0.165**	0.143**	0.119**	0.090*	0.104**	0.094*	0.143*	0.141**	0.140**	0.115**	0.089*	0.105**	0.149**
Children ≤ 7 (*n *= 103)	0.027	0.068	0.014	0.034	0.038	0.057	0.132	− 0.010	− 0.011	− 0.054	− 0.132	0.083	0.107
Children 8–10 (*n *= 253)	0.260**	0.236**	0.245**	0.176**	0.128*	0.163*	0.203**	0.208**	0.200**	0.185**	0.162*	0.166**	0.158*
Children ≥ 11 (*n *= 300)	0.086	0.040	0.007	0.000	0.029	0.040	0.068	0.097	0.114*	0.099	0.072	0.014	0.119*

	CY-BOCS total score	CY-BOCS obsessions	CY-BOCS obsession dimensions					CY-BOCS compulsions	CY-BOCS compulsion dimensions				
		Subscale score	Time	Interference	Distress	Resistance	Control	Subscale score	Time	Interference	Distress	Resistance	Control
Total sample (*n *= 656)	0.083	0.106**	0.093*	0.072	0.138**	0.079*	0.090*	0.126**	0.129**	0.118**	0.152**	0.099*	0.079*
Children ≤ 7 (*n *= 103)	0.026	0.070	0.108	0.072	0.102	0.046	0.004	0.072	0.099	0.106	0.140	0.015	0.016
Children 8–10 (*n *= 253)	0.178	0.184**	0.162*	0.138*	0.211**	0.129*	0.178**	0.221**	0.220**	0.204**	0.238**	0.120	0.196**
Children ≥ 11 (*n *= 300)	0.044	0.030	0.006	− 0.003	0.061	0.034	0.032	0.033	0.032	0.029	0.046	0.073	− 0.009

*PUTS* Premonitory Urge for Tics Scale using item 1–9 [12], *YGTSS* Yale Global Tic Severity Scale [15], *CY*-*BOCS* Children’s Yale-Brown Obsessive–Compulsive Scale [18]

Pearson *r* correlations *p < 0.05; **p < 0.001

A similar pattern appeared for CY-BOCS subscale scores with small but significant positive correlations with the PUTS in the total sample, which were again driven by children aged 8–10 years. Although the CY-BOCS obsession and compulsion subscales reached statistical significance, correlations with the CY-BOCS total score did not (see Table 3 for the results of the three-group analysis, and Supplementary Table S2b for the results of the two-group analysis).

After removing the two items that are often associated with OCD symptomatology in the three age groups; i.e. ‘the feeling that something is not “just right”’ and ‘the feeling that something is not complete’ (items 4 and 5), the significant correlations between PUTS severity and OCD severity disappeared for the obsessions-subscale and diminished for the compulsions-subscale (Supplementary Table S2c).

### Associations of the PUTS with other symptom domains

A similar age-related pattern was observed after correlating the PUTS total score with scores for ASD, ADHD, ODD, and internalizing and externalizing behaviors for the total sample and for the three age groups (see Supplementary Table S2d). Significant positive, yet weak, correlations between the PUTS total score and measures for ADHD, internalizing, and externalizing behaviors were only present in children aged 8–10, but not in younger or older children. Other correlations did not yield significant results.

### Exploratory factor analysis

See Table 4 for the factor loadings of the PUTS for the total sample, and divided by the three age groups (and Supplementary Table S2f for the factor loadings of the PUTS for children ≤ 10 years). The inter-item correlation matrix (Supplementary Table S2e) showed good factorability of the PUTS in all groups, except for item 1, which was removed from the factor analysis in all groups due to multiple low inter-item-correlations (*r* < 0.20). Similarly, for children ≥ 11 years, items 2 and 9 were removed (Supplementary Tables S2e, S2f). There was no multicollinearity between PUTS items.

**Table 4:** Factor loadings and communalities based on an exploratory factor analysis with oblimin rotation for the PUTS

	Total sample (*n *= 656)		≤ 7 years (*n *= 103)		8–10 years (*n *= 253)		≥ 11 years (*n *=300)				
	Factor 1	Comm	Factor 1	Comm	Factor 1	Comm	Pattern matrix		Structure matrix		Comm
							Factor 1	Factor 2	Factor 1	Factor 2	
1. Right before I do a tic, I feel like my insides are itchy											
2. Right before I do a tic, I feel pressure inside my brain or body	0.55	0.30	0.49	0.26	0.66	0.44					
3. Right before I do a tic, I feel “wound up” or tense inside	0.51	0.26			0.49	0.24		0.47		0.38	0.23
4. Right before I do a tic, I feel like something is not “just right”	0.51	0.26	0.51	0.34	0.57	0.32	0.49		0.73		0.53
5. Right before I do a tic, I feel like something is not complete	0.51	0.26	0.48	0.30	0.54	0.29	0.34		0.60		0.36
6. Right before I do a tic, I feel like there is energy in my body that needs to get out	0.56	0.31	0.67	0.48	0.56	0.32					
7. I have these feelings almost all the time before I do a tic	0.77	0.59	0.85	0.69	0.73	0.53		0.87		0.89	0.75
8. These feelings happen for every tic I have	0.66	0.43	0.75	0.51	0.59	0.35		0.86		0.79	0.62
9. After I do the tic, the itchiness, energy, pressure, tense feelings, or feelings that something is not ‘‘just right’’ or complete go away, at least for a little while	0.57	0.32	0.55	0.35	0.62	0.38					
% of variance	42.2		47.4		43.7		47.4	20.5			
Kaiser–Meyer–Olkin (KMO)	0.84		0.84		0.85		0.68				

Due to weak inter-item correlation item 1 was removed a priori from the factor analysis for the total sample, children ≤ 10 years, and children between 8 and 10 years, while items 1, 2 and 9 were removed for children ≥ 11 years; In addition, due to low communality of < 0.2, item 3 was removed from the factor analysis for children ≤ 7 years, and item 6 for children ≥ 11 years. For the one-factor solutions, we report the factor loadings of the unrotated matrix. For the two-factor solution (only for children ≥ 11 years), we report the factor loadings from the pattern matrix and the structure matrix

*PUTS* Premonitory Urge for Tics Scale using item 1–9 [12], *Comm* communalities

After removing item 1 from the respective groups, the KMO for the total sample and all age groups was above the recommended value of 0.6 indicating sufficient sampling adequacy (Table 4 [34]). Furthermore, Bartlett’s test of sphericity was significant for all groups [i.e., the total sample: *χ*^2^(28) = 1306.6, *p *< 0.001; children ≤ 7 years *χ*^2^(28) = 207.7 *p *< 0.001; children between 8 and 10 years: *χ*^2^(28) = 535.8, *p *< 0.001; children ≤ 10 years: *χ*^2^(28) = 751.5, *p *< 0.001; and children ≥ 11 years: *χ*^2^(15) = 345.6, *p *< 0.001, respectively], indicating that correlations between items were sufficiently large to conduct an EFA.

An EFA with oblimin rotation across PUTS items 2–9 for the total sample indicated one factor (see Table 4). Initial eigenvalues demonstrated that this factor explained 42.2% of the variance. In the three-group analysis, an EFA across items 2–9 for children ≤ 7 years also revealed that all items loaded on one factor, explaining 47.4% of the variance (see Table 4), while it explained 43.7% for children between 8 and 10 years. In addition, in the two-group analysis, all items loaded on one factor for children ≤ 10 years, explaining 44% of the variance (Supplementary Table S2f). However, an exploratory factor analysis for children ≥ 11 years in both analyses revealed two factors, with a total explained variance of 67.9%. Notably, the first factor that explained the most variance in the two-factor-solution included two OCD-related items (items 4 and 5). In children ≥ 11 years, item 6 (‘the feeling of an energy that needs to get out’) had a communality score of .18, while in children ≤ 10 years item 3 (‘Right before I do a tic, I feel ‘‘wound up’’ or tense inside’) had a communality score of .17, thus these items were subsequently removed from the respective factor analyses. Finally, after removing the two OCD-related items from all analyses, only one-factor solutions emerged for all groups. As a final remark, when repeating all analyses with the outliers included, all results remained similar.

## Discussion

The present study investigated the psychometric properties of the PUTS in 656 children and adolescents aged 3–16 years. Contrary to previous smaller sized studies [12, 16, 17] that reported insufficient psychometric properties of the PUTS in children younger than 11 years, our results showed satisfactory reliability also in younger children. This suggests that the PUTS is suitable for children and adolescents across a broad age range. We found that the PUTS correlated significantly, yet weakly, with tic and OCD symptom severity, and with measures for ADHD and internalizing and externalizing behaviors, specifically in children between 8 and 10 years. These overall weak correlations point to different constructs as assessed by the PUTS and other scales measuring symptoms of different clinical diagnoses. While the PUTS was originally designed as a one-dimensional measure, we observed an underlying two-factor structure of the PUTS in children and adolescents above 10 years. This pointed to two distinct dimensions that are measured by the PUTS, of which one factor contained the two items that previously has been associated with OCD (i.e. ‘the feeling that something is not “just right” or not complete’). Consistent with Woods et al. [12], PUTS item number 10 (measuring the ability to stop tics even if only for a short period of time) correlated less with the rest of the scale compared to the other items and, therefore, should not be used as part of the questionnaire for all age groups.

Internal reliability for all investigated age groups was in the good to excellent range. Previous authors explained their findings of low internal reliability of the PUTS in children of 11 and younger by difficulties in recognizing or articulating awareness of the urge [12, 16]. It has also been suggested that perhaps the urges are not present during the initial stages of TS, but develop on average a few years after the first onset of tics, which usually is around 6 years of age [3, 10, 38]. While our study confirms tic onset around 6 years of age, we also observed that 80 to 95% of the children of 10 years and younger experienced urges to some extent, which is more than previously reported in a large pediatric sample (47% in children under the age of 10 [14]). Yet, our findings are similar to Woods et al. [12], who originally reported that all children of 10 years and younger experienced premonitory urges. Our study suggests that the presence of premonitory urges may already exist about the time tics develop and that urges can be reliably identified early in development. Additional support for the early presence of premonitory urges stems from the demonstrated efficacy of behavioral treatment focusing on premonitory urges in children under the age of 10 [39]. However, we did observe an age-dependent increased awareness of the premonitory urge across the age groups, with the youngest children reporting the least amount of urges (81%) and the oldest participants the most (97.5%). It remains questionable to what extent very young children are able to reliably fill in a self-report questionnaire. We cannot exclude that the parents have assisted in answering the PUTS items, even though there are reports of 5-year-olds reliably filling in age-appropriate health-related questionnaires [40]. In sum, although our results point to a reliable use of the PUTS from young childhood well into adolescence, more research is warranted to further explore the possible existence and reporting of premonitory urges in very young children.

The weak, and largely non-existent correlations between the PUTS and tic severity as assessed by (subscales of) the YGTSS were unexpected. If tics are indeed semi-voluntary responses to premonitory urges [23], which is also presumed by one of the most endorsed items of the PUTS in our study (i.e., item 9, ‘the feelings go away after I do the tic’), then more severe urges would be expected to be related to more severe tics. Our results are in contrast to a recent meta-analysis observing a moderate correlation (*r *= 0.296) between the severity of premonitory urges and tic symptoms [44], although this was based on a small number of studies using relatively small samples (*n *= 40–122) across children and adults, which may have biased findings [41]. One explanation for the weak association between premonitory urges and tic severity in our study may be that the PUTS and YGTSS questionnaires are actually measuring different constructs relating to distinct phenomena. This is in line with Ganos et al. [42] who suggested distinct neurological pathways for premonitory urges, tic generation, and tic suppression; and that premonitory urges may not be the driving force behind tics [43]. A similar distinction has previously been mentioned by Brandt et al. [35], showing only a weak relationship between premonitory urges measured by a real-time urge monitor and tic frequency; a relation that even weakened during tic suppression, suggesting a decoupling of urges and tics. On another note, limitations of the PUTS have been recognized before (e.g. being designed as a unitary construct, and not allowing the respondent to distinguish between specific urges for different tics [45]), leading to the recent development of a new measure to assess premonitory urges (I-PUTS, [45]). However, more research is warranted to investigate the validity of this new measure in comparison with the PUTS. Regarding the age effects, perhaps younger children are less able to distinguish between urges and tics [10], whereas the ability to differentiate between these phenomena may become more pronounced with increasing age. In children and adolescents above 10 years on the other hand, more severe urges may not necessarily be accompanied by more severe tics, as indicated in our study by the disappearing relation between the severity of urges and tics, perhaps due to a better awareness of the urges.

Two items of the PUTS representing mental phenomena that may be considered part of the OCD spectrum (i.e., items 4 and 5 referring to feelings of not “just right” and not complete) largely drove the association for children between 8 and 10 years in our study; this may suggest that a relation between the PUTS and OCD symptoms is spurious. Mixed findings regarding associations between PUTS severity and OCD severity have been documented before [12, 17, 23], although these results were only found in children of 11 years and older and in adults, while the recent meta-analysis that included these studies indicated a moderate association between premonitory urges and obsessive–compulsive symptoms [44]. Why this association exclusively existed in children between 8 and 10 years in our study cannot be readily explained, as no differences in OCD symptom severity between the investigated age groups were observed. Perhaps children between 8 and 10 years, at an age when symptoms of OCD are typically developing [37], have difficulty differentiating between premonitory urges that are associated with tics and those associated with OCD symptoms, which may become easier with increasing age [36]. Alternatively, as the frequency of these two mental urges appeared to slightly increase with age, so did other items captured by the PUTS, possibly outweighing the influence of these OCD-like urges, explaining the lack of association between the PUTS and OCD symptoms in children of 11 years and older. Of note, even though the correlations between premonitory urges and OCD symptoms in children between 8 and 10 years were significant, they were small, similar to the other age groups, indicating a weak relationship. Further research is needed to elucidate the complex relationship between tic and OCD-related urges across development.

Consistent with the original PUTS [12], we found a one-factor solution in children of 10 years and younger. Confirming recent studies in children and adults [16, 19], and in line with the above discussed results, we found support for a two-factor solution in children of 11 years and older. The first and most important factor, explaining the most variance, pointed to items that are typically associated with obsessive–compulsive symptoms [5], which suggest a distinction between sensory phenomena related to OCD and those related to tics. The second factor, which explained less variance, included items that addressed the ‘frequency of urges’ before a tic in children as of 11 years (i.e., ‘if the feelings are present almost all the time before a tic’ and if ‘these feelings happen for every tic’). This is in line with Raines [16] and has similarities to Brandt et al.’s [19] second factor described as the ‘overall intensity of urges’. In sum, the age-related differences we observed so far regarding the underlying structure (one versus two-factor solution) of the PUTS, and the various items that had to be removed from the analyses in the older age group may indicate that the questions of the PUTS may be differently perceived at various ages.

A major strength of this study was the large sample size and wide age range, allowing us to explore age-dependency across a broad age range. Potential limitations were, first, the use of multiple clinical sites across Europe, reflecting possible site differences in scoring and clinical populations. By regressing out the effect of site per variable, we tried to account for this bias. In addition, clinical interviewers were regularly trained and standardization of the procedures was discussed bi-annually. Second, our sample showed a relatively low number of comorbid ADHD and OCD diagnoses compared to previous studies investigating the psychometric properties of the PUTS [12, 16, 17], perhaps indicating a less severely affected sample.

In conclusion, the PUTS questionnaire exhibits good internal reliability in children and adolescents, also in children under the age of 10, which is younger than previously thought. Our study indicates that premonitory urges appear to be present at an early age, possibly starting at the onset of tics in some children. The overall weak correlations between the PUTS and, respectively, YGTSS and CY-BOCS scores suggest that different constructs are measured by the respective scales, distinguishing between premonitory urges, tics, and obsessive–compulsive symptoms. The observed two-factor structure of the PUTS in children of 11 years and older indicates that two separate dimensions of premonitory urges are measured in this age group, distinguishing between sensory phenomena related to tics and mental phenomena as often found in OCD. The age-related differences observed in this study may indicate the need for the development of an age-specific questionnaire to asses urges. Future research should focus on a closer examination of the use of the PUTS across development and how well it captures possible age-dependent differences in premonitory urges and the relation with tics and comorbid symptoms.

## Electronic supplementary material

Below is the link to the electronic supplementary material.

Supplementary material 1 (DOCX 50 kb)
