# Supplementary material for: The Premonitory Urge for Tics Scale in a large sample of children and adolescents: psychometric properties in a developmental context. An EMTICS study
Source: Eur Child Adolesc Psychiatry. 2019 Dec 4;29(10):1411–24. doi: 10.1007/s00787-019-01450-1 (PMC7501098; doi:10.1007/s00787-019-01450-1)
Supplement: Supplementary file 1 — Supplementary material 1 (DOCX 50 kb) [file 787_2019_1450_MOESM1_ESM.docx]

**Supplemental Material**

**Title:** The Premonitory Urge for Tics Scale in a large sample of children and adolescents: Psychometric properties in a developmental context. An EMTICS study

**Journal name:** European Child & Adolescent Psychiatry

**Authors**: Thaïra J.C. Openneer^a^, Zsanett Tarnok^b^, Emese Bognar^b^, Noa Benaroya-Milshtein^c^, Blanca Garcia Delgar^d^, Astrid Morer^d,e,f^, Tami Steinberg^c^, Pieter J. Hoekstra^a*^, Andrea Dietrich^a*^, and the EMTICS collaborative group

**Affiliations:**

^a^University of Groningen, University Medical Center Groningen, Department of Child and Adolescent Psychiatry, Groningen, The Netherlands

^b^Vadaskert Child and Adolescent Psychiatric Hospital, Budapest, Hungary

^c^Child and Adolescent Psychiatry Department, Schneider Children's Medical Center of Israel, affiliated to Sackler Faculty of Medicine, Tel Aviv University, Petah-Tikva, Israel
^d^Department of Child and Adolescent Psychiatry and Psychology, Institute of Neurosciences, Hospital Clinic Universitari, Barcelona, Spain
^e^Institut d'Investigacions Biomediques August Pi i Sunyer (IDIBAPS), Barcelona, Spain

^f^Centro de Investigacion en Red de Salud Mental (CIBERSAM), Instituto Carlos III, Spain

*Contributed equally

**Corresponding author:** Address correspondence to Thaïra J.C. Openneer, University of Groningen, University Medical Center Groningen, Department of Child and Adolescent Psychiatry, Hanzeplein 1 XA10, 9713 GZ Groningen, The Netherlands; E-mail: [t.openneer@accare.nl](mailto:t.openneer@accare.nl).

**Supplement 1 Measures of ADHD, ODD, ASD and internalizing and externalizing disorders**

*The Swanson Nolan and Pelham-IV rating scale (SNAP-IV).* The SNAP-IV [1,2] (Cronbach's alpha in our study α=.95) is a validated 26-item parent-rated questionnaire, measuring ADHD symptoms (18 items; 9 for inattentive, 9 for hyperactive/impulsive) and ODD symptoms (8 items), on a four-point scale. To assess ADHD and ODD severity, sum scores were calculated for respectively ADHD (first 18 items, range 0-54, α=.94) and ODD (last 8 items, range 0-24, α=.91), with a higher score indicating higher symptom severity.

*Autism Spectrum Screening Questionnaire (ASSQ).* The ASSQ [3] is a validated 27-item screening checklist measuring autism-related symptoms on a three-point scale (Cronbach's alpha in our study α=.91). The items cover impairments in social interactions (11 items), restricted and repetitive behavior (5 items), communication problems (6 items), and motor clumsiness and associated symptoms (5 items). A total sum score of all items (range 0-54) was calculated to assess ASD severity, with higher scores indicating higher symptom severity.

*Strengths and Difficulties Questionnaire (SDQ).* The SDQ [4] is a widely used parent-rated screening instrument (Cronbach's alpha in our study α=.95), covering 25 items on 5 subscales, including emotional problems, conduct, hyperactivity, peer problems, and social behavior over the last two weeks. A severity score for internalizing problems was calculated by summing up the emotional problems and peer problems subscales (10 items, range 0-20; α=.77), while severity of externalizing behaviors was calculated by adding the hyperactivity and conduct subscales (10 items, range 0-20; α=.80). A higher score indicated higher severity of internalizing and externalizing symptoms.

**Supplement 2 Additional Tables**

**Table S2a** Comparison of individual PUTS items between children ≤ 10 years (*n* = 356) and children ≥ 11 years (*n* = 300): Means, standard deviations, item-total correlations (Pearson’s *r*) and internal reliability (Cronbach’s *α*)

|  | Children ≤ 10 years | | | | Children ≥ 11 years | | | | Test statistic |
| --- | --- | --- | --- | --- | --- | --- | --- | --- | --- |
|  | Mean | SD | Pearson’s *r* | α if item removed | Mean | SD | Pearson’s *r* | α if item removed |  |
|  |  |  |  |  |  |  |  |  |  |
| PUTS 1 | 1.57 | .90 | .42** | .81 | 1.64 | .95 | .20** | .74 | *T*(655)=-1.08 |
| PUTS 2 | 1.62 | .94 | .55** | .80 | 1.87 | 1.01 | .42** | .71 | *T*(654)=-3.30* |
| PUTS 3 | 1.77 | .98 | .48** | .80 | 2.06 | 1.00 | .42** | .71 | *T*(634)=-3.67** |
| PUTS 4 | 1.62 | .94 | .47** | .81 | 1.76 | 1.01 | .41** | .71 | *T*(655)=-1.77 |
| PUTS 5 | 1.52 | .90 | .45** | .81 | 1.72 | 1.04 | .43** | .71 | *T*(595)=-2.61* |
| PUTS 6 | 2.00 | 1.11 | .54** | .80 | 2.38 | 1.10 | .42** | .71 | *T*(654)=-4.42** |
| PUTS 7 | 2.10 | 1.10 | .72** | .78 | 2.59 | 1.11 | .62** | .68 | *T*(652)=-5.69** |
| PUTS 8 | 1.94 | 1.08 | .57** | .80 | 2.28 | 1.14 | .53** | .69 | *T*(630)=-3.88** |
| PUTS 9 | 2.31 | 1.21 | .61** | .79 | 2.66 | 1.15 | .44** | .71 | *T*(648)=-3.81** |
| PUTS 10 | 2.33 | 1.01 | .26** | .84 | 3.00 | 1.00 | .06 | .76 | *T*(642)=-6.90** |
| *α* 9-items | .84 | | | | .76 | | | |  |
| *α* 10-items | .80 | | | | .72 | | | |  |

PUTS, Premonitory Urge for Tics Scale item [5]; each item scored on a 4-point scale from 1 = ‘not at all true’ to 4 = ‘very much true’; .*α* 9-items indicated the Cronbach’s *α* for item 1-9 of the PUTS, whereas *α* 10-items indicates the Cronbach’s *α* for item 1-10 of the PUTS; Between-group differences were tested by an independent T-test; ** p*<.05; ** *p*<.001

**Table S2b** Correlations between the PUTS total score and respective YGTSS and CY-BOCS scales for children ≤ 10 years (*n* = 356) and children ≥ 11 years (*n* = 300)

|  | **YGTSS total score** | **YGTSS motor tics** | **YGTSS motor tic dimensions** | | | | | **YGTSS vocal tics** | **YGTSS vocal tic dimensions** | | | | | | | | |
| --- | --- | --- | --- | --- | --- | --- | --- | --- | --- | --- | --- | --- | --- | --- | --- | --- | --- |
|  |  | Subscale score | Number | Frequency | Intensity | Complexity | Interference | Subscale score | Number | Frequency | | Intensity | | Complexity | | | Interference |
| Children ≤ 10 years | .201** | .204** | .192** | .145** | .129* | .139** | .200** | .148** | .143** | | .109* | .085 | | .145** | | | .148** |
| Children ≥ 11 years | .086 | .040 | .007 | .000 | .029 | .040 | .068 | .097 | .114* | | .099 | .072 | | .014 | | | .119* |
|  | **CY-BOCS total score** | **CY-BOCS obsessions** | **CY-BOCS obsession dimensions** | | | | | **CY-BOCS compulsions** | **CY-BOCS compulsion dimensions** | | | | | | | | |
|  |  | Subscale score | Time | Interference | Distress | Resistance | Control | Subscale score | Time | Interference | | | Distress | | Resistance | Control | |
| Children ≤ 10 years | .133 | .158** | .157** | .122* | .194** | .109* | .132* | .186** | .201** | .180** | | | .215** | | .083 | .132** | |
| Children ≥ 11 years | .044 | .030 | .006 | -.003 | .061 | .034 | .032 | .033 | .032 | .029 | | | .046 | | .073 | -.009 | |

PUTS, the 9-item (item 1-9) Premonitory Urge for Tics Scale [5]; YGTSS, Yale Global Tic Severity Scale [6]; CY-BOCS, Children’s Yale-Brown Obsessive-Compulsive Scale [7]; Pearson *r* correlations **p*<.05; ***p*<.001

**Table S2c** Correlations between the PUTS total score without the two OCD-related items 4 and 5, and CY-BOCS scales for the total sample and different age groups

|  | **CY-BOCS total score** | **CY-BOCS obsessions** | **CY-BOCS obsession dimensions** | | | | | **CY-BOCS compulsions** | **CY-BOCS compulsion dimensions** | | | | |
| --- | --- | --- | --- | --- | --- | --- | --- | --- | --- | --- | --- | --- | --- |
|  |  | Subscale score | Time | Interference | Distress | Resistance | Control | Subscale score | Time | Interference | Distress | Resistance | Control |
| Total sample (*n* = 656) | .036 | .041 | .043 | .011 | .074 | .029 | .026 | .071 | .078 | .070 | .100* | .066 | .026 |
| Children =<7 (*n* = 103) | -.060 | .012 | .053 | .018 | .042 | -.001 | -.032 | .025 | .044 | .083 | .119 | -.046 | -.024 |
| Children 8-10 (*n* = 253) | .107 | .087 | .072 | .046 | .121 | .061 | .084 | .146* | .132* | .141* | .127* | .087 | .121 |
| Children =>11 (*n* = 300) | .019 | -.024 | -.022 | -.057 | .005 | -.011 | -.024 | -.020 | .002 | -.027 | -.005 | .041 | -.067 |

PUTS, Premonitory Urge for Tics Scale using item 1,2,3,6,7,8 and 9 [5]; CY-BOCS, Children’s Yale-Brown Obsessive-Compulsive Scale [7]; Pearson *r* correlations **p*<.05

**Table S2d** Pearson *r* correlations between the PUTS total score and respective severity scores of comorbid problems for the total sample and different age groups

|  | **ASD** | **ADHD** | **ODD** | **INT** | **EXT** |
| --- | --- | --- | --- | --- | --- |
| Total sample (*n* = 656) | .025 | .080* | .024 | .084* | .047 |
| Children ≤ 7 years (*n* = 103) | .011 | .047 | .034 | -.073 | .024 |
| Children 8-10 years (*n* = 253) | .092 | .140* | .100 | .177** | .149* |
| Children ≥ 11 years (*n* = 300) | -.060 | .019 | -.049 | -.003 | -.052 |

PUTS, the 9-item (item 1-9) Premonitory Urge for Tics Scale [5]; Autism spectrum disorder (ASD) symptom severity was measured by the Autism Spectrum Screening Questionnaire (ASSQ [3]); attention-deficit/hyperactivity disorder (ADHD) and oppositional defiant disorder (ODD) symptom severity by the Swanson Nolan and Pelham-IV rating scale (SNAP-IV [1]); and internalizing (INT) and externalizing (EXT) symptoms by the Strengths and Difficulties Questionnaire (SDQ [4]) **p*<.05; ***p*<.001.

**Table S2e** Inter-item correlation matrix for 9 PUTS items for the total sample and different age groups

| **Total sample (*n* = 656)** | | | | | | | | | | | | | |
| --- | --- | --- | --- | --- | --- | --- | --- | --- | --- | --- | --- | --- | --- |
|  | PUTS 1 | PUTS 2 | PUTS 3 | PUTS 4 | PUTS 5 | PUTS 6 | | | PUTS 7 | | PUTS 8 | | PUTS 9 |
| PUTS 1 | - |  |  |  |  |  | | |  | |  | |  |
| PUTS 2 | .25** | - |  |  |  |  | | |  | |  | |  |
| PUTS 3 | .15** | .34** | - |  |  |  | | |  | |  | |  |
| PUTS 4 | .15** | .28** | .33** | - |  |  | | |  | |  | |  |
| PUTS 5 | .13** | .28** | .31** | .45** | - |  | | |  | |  | |  |
| PUTS 6 | .19** | .38** | .34** | .24** | .26** | - | | |  | |  | |  |
| PUTS 7 | .31** | .38** | .38** | .36** | .33** | .43** | | | - | |  | |  |
| PUTS 8 | .24** | .31** | .31** | .26** | .30** | .37** | | | .67** | | - | |  |
| PUTS 9 | .22** | .32** | .28** | .30** | .29** | .30** | | | .48** | | .37** | | - |
| **Children ≤ 7 years (*n* = 103)** | | | | | | |  |  | |  | |  | |
|  | PUTS 1 | PUTS 2 | PUTS 3 | PUTS 4 | PUTS 5 | PUTS 6 | | | PUTS 7 | | PUTS 8 | | PUTS 9 |
| PUTS 1 | - |  |  |  |  |  | | |  | |  | |  |
| PUTS 2 | .43** | - |  |  |  |  | | |  | |  | |  |
| PUTS 3 | .37** | .37** | - |  |  |  | | |  | |  | |  |
| PUTS 4 | .03 | .26** | .30** | - |  |  | | |  | |  | |  |
| PUTS 5 | .11 | .21* | .26** | .37** | - |  | | |  | |  | |  |
| PUTS 6 | .24* | .43** | .37** | .33** | .33** | - | | |  | |  | |  |
| PUTS 7 | .35** | .34** | .38** | .52** | .44** | .56** | | | - | |  | |  |
| PUTS 8 | .30** | .36** | .22* | .38** | .39** | .51** | | | .68** | | - | |  |
| PUTS 9 | .21* | .31** | .30** | .27** | .35** | .35** | | | .49** | | .34** | | - |
| **Children 8-10 years (*n* = 253)** | | | | | | |  |  | |  | |  | |
|  | PUTS 1 | PUTS 2 | PUTS 3 | PUTS 4 | PUTS 5 | PUTS 6 | | | PUTS 7 | | PUTS 8 | | PUTS 9 |
| PUTS 1 | - |  |  |  |  |  | | |  | |  | |  |
| PUTS 2 | .24** | - |  |  |  |  | | |  | |  | |  |
| PUTS 3 | .21** | .36** | - |  |  |  | | |  | |  | |  |
| PUTS 4 | .26** | .40** | .34** | - |  |  | | |  | |  | |  |
| PUTS 5 | .19** | .39** | .33** | .49** | - |  | | |  | |  | |  |
| PUTS 6 | .19** | .41** | .36** | .26** | .28** | - | | |  | |  | |  |
| PUTS 7 | .40** | .49** | .38** | .39** | .28** | .43** | | | - | |  | |  |
| PUTS 8 | .34** | .34** | .31** | .24** | .27** | .32** | | | .60** | | - | |  |
| PUTS 9 | .31** | .36** | .33** | .35** | .33** | .37** | | | .53** | | .40** | | - |

Table continues next page.

| **Children ≤ 10 years (*n* = 356)** | | | | | | | | | |  |  |
| --- | --- | --- | --- | --- | --- | --- | --- | --- | --- | --- | --- |
|  | PUTS 1 | PUTS 2 | PUTS 3 | PUTS 4 | PUTS 5 | PUTS 6 | PUTS 7 | PUTS 8 | | | PUTS 9 |
| PUTS 1 | - |  |  |  |  |  |  |  | | |  |
| PUTS 2 | .29** | - |  |  |  |  |  |  | | |  |
| PUTS 3 | .25** | .37** | - |  |  |  |  |  | | |  |
| PUTS 4 | .19** | .36** | .32** | - |  |  |  |  | | |  |
| PUTS 5 | .18** | .34** | .31** | .45** | - |  |  |  | | |  |
| PUTS 6 | .22** | .42** | .37** | .28** | .30** | - |  |  | | |  |
| PUTS 7 | .40** | .45** | .39** | .42** | .33** | .47** | - |  | | |  |
| PUTS 8 | .33** | .35** | .30** | .28** | .31** | .38** | .63** | - | | |  |
| PUTS 9 | .29** | .35** | .33** | .33** | .34** | .37** | .52** | .40** | | | - |
| **Children ≥ 11 years (*n* = 300)** | | | | | | | | |  |  | |
|  | PUTS 1 | PUTS 2 | PUTS 3 | PUTS 4 | PUTS 5 | PUTS 6 | PUTS 7 | PUTS 8 | | | PUTS 9 |
| PUTS 1 | - |  |  |  |  |  |  |  | | |  |
| PUTS 2 | .19** | - |  |  |  |  |  |  | | |  |
| PUTS 3 | .03 | .29** | - |  |  |  |  |  | | |  |
| PUTS 4 | .20* | .17** | .31** | - |  |  |  |  | | |  |
| PUTS 5 | .08 | .20** | .27** | .43** | - |  |  |  | | |  |
| PUTS 6 | .20* | .31** | .24** | .20** | .20** | - |  |  | | |  |
| PUTS 7 | .22** | .27** | .34** | .27** | .30** | .34** | - |  | | |  |
| PUTS 8 | .14* | .26** | .29** | .23** | .26** | .33** | .68** | - | | |  |
| PUTS 9 | .14* | .26** | .20** | .25** | .21** | .18** | .39** | .31** | | | - |

PUTS, Premonitory Urge for Tics Scale using item 1-9 [5]; ***p*<.001 **p*<.05

**Table S2f** Factor loadings and communalities based on an exploratory factor analysis for the PUTS

|  | Children ≤ 10 years  (*n* = 356) | |
| --- | --- | --- |
|  |  |  |
|  | Factor 1 | Communalities |
|  |  |  |
| 1. Right before I do a tic, I feel like my insides are itchy. |  |  |
| 2. Right before I do a tic, I feel pressure inside my brain or body. | .62 | .38 |
| 3. Right before I do a tic, I feel ‘‘wound up’’ or tense inside | .48 | .23 |
| 4. Right before I do a tic, I feel like something is not ‘‘just right.’’ | .55 | .30 |
| 5. Right before I do a tic, I feel like something isn’t complete. | .53 | .28 |
| 6. Right before I do a tic, I feel like there is energy in my body that needs to get out. | .60 | .36 |
| 7. I have these feelings almost all the time before I do a tic. | .76 | .58 |
| 8. These feelings happen for every tic I have. | .63 | .39 |
| 9. After I do the tic, the itchiness, energy, pressure, tense feelings, or feelings that something isn’t ‘‘just right’’ or complete go away, at least for a little while. | .61 | .37 |
| % of variance | 44.0 |  |
| Kaiser-Meyer-Olkin (KMO) | .86 |  |

PUTS, Premonitory Urge for Tics Scale using item 1-9 [5]; Due to weak inter-item-correlation item 1 was removed a priori from the factor analysis for children ≤ 10, see Table 4 for the factor analysis for children ≥ 11 years. Values reported are the factor loadings from the unrotated matrix.

**References supplemental material**

1. Swanson JM (1992) School-based assessments and interventions for ADD students. Irvine, CA, K. C. Publishing.

2. Bussing R, Fernandez M, Harwood M, Wei Hou W, Garvan CW, Eyberg SM, et al (2008) Parent and teacher SNAP-IV ratings of attention deficit/hyperactivity disorder symptoms: Psychometric properties and normative rating from a school district sample. Assessment 15(3):317–328.

3. Ehlers S, Gillberg C, Wing L (1999) A screening questionnaire for Asperger syndrome and other high-functioning autism spectrum disorders in school age children. J Autism Dev Disord 29(2):129–141.

4. Goodman R (1997) The Strengths and Difficulties Questionnaire: A research note. J Child Psychol Psychiatry 38(5):581–586.

5. Woods DW, Piacentini J, Himle MB, Chang S (2005) Premonitory Urge for Tics Scale (PUTS): Initial psychometric results and examination of the premonitory urge phenomenon in youths with tic disorders. J Dev Behav Pediatr 26(6):397–403.

6. Leckman JF, Riddle MA, Hardin MT, Ort SL, Swartz KL, Stevenson J, Cohen DJ (1989) The Yale Global Tic Severity Scale: Initial testing of a clinician-rated scale of tic severity. J Am Acad Child Adolesc Psychiatry 28:566–573.

7. Scahill L, Riddle MA, McSwiggin-Hardin M, Ort SI, King RA, Goodman WK, et al (1997) Children’s Yale-Brown Obsessive Compulsive Scale: Reliability and validity. J Am Acad Child Adolesc Psychiatry 36(6):844–852.
